# Investigating the predictive value of different resting-state functional MRI parameters in obsessive-compulsive disorder

**DOI:** 10.1038/s41398-018-0362-9

**Published:** 2019-01-17

**Authors:** Xuan Bu, Xinyu Hu, Lianqing Zhang, Bin Li, Ming Zhou, Lu Lu, Xiaoxiao Hu, Hailong Li, Yanchun Yang, Wanjie Tang, Qiyong Gong, Xiaoqi Huang

**Affiliations:** 1 Huaxi MR Research Center (HMRRC), Department of Radiology, West China Hospital of Sichuan University, 37 Guo Xue Xiang, Chengdu, Sichuan 610041 China; 2 Mental Health Center, Department of Psychiatry, West China Hospital of Sichuan University, 37 Guo Xue Xiang, Chengdu, Sichuan 610041 China

## Abstract

Previous resting-state functional magnetic resonance imaging (rs-fMRI) studies of obsessive-compulsive disorder (OCD) have facilitated our understanding of OCD pathophysiology based on its intrinsic activity. However, whether the group difference derived from univariate analysis could be useful for informing the diagnosis of individual OCD patients remains unclear. We aimed to apply multivariate pattern analysis of different rs-fMRI parameters to distinguish drug-naive patients with OCD from healthy control subjects (HCS). Fifty-four drug-naive OCD patients and 54 well-matched HCS were recruited. Four different rs-fMRI parameter maps, including the amplitude of low-frequency fluctuations (ALFF), fractional amplitude of low-frequency fluctuations (fALFF), regional homogeneity (ReHo) and functional connectivity strength (FCS), were calculated. Training of a support vector machine (SVM) classifier using rs-fMRI maps produced voxelwise discrimination maps. Overall, the classification accuracies were acceptable for the four rs-fMRI parameters. Excellent performance was achieved when ALFF maps were employed (accuracy, 95.37%, *p* < 0.01), good performance was achieved by using ReHo maps, weaker performance was achieved by using fALFF maps, and fair performance was achieved by using FCS maps. The brain regions showing the greatest discriminative power included the prefrontal cortex, anterior cingulate cortex, precentral gyrus, and occipital lobes. The application of SVM to rs-fMRI features may provide potential power for OCD classification.

## Introduction

Obsessive-compulsive disorder (OCD) is a common debilitating disorder characterized by persistent intrusive thoughts and repetitive actions, with a prevalence of 1% to 3%^1^. While cortico–striato–thalamo–cortical (CSTC) dysfunction has been found to contribute to the pathogenesis of OCD, emerging evidence suggests that broader brain regions, such as the parietal cortex and insula, are involved in this disorder^2–4^. Such widespread alterations may be due to the diversity of tasks used to investigate OCD. The investigation of altered patterns of brain activity during rest in OCD has the advantage of identifying neural mechanisms that are not specific to a task, which will provide a reliable measure of baseline brain activity^5^ and may complement and extend findings from task-based studies.

Recent advances in resting-state functional magnetic resonance imaging (rs-fMRI) have facilitated our understanding of OCD pathophysiology based on its intrinsic activity. Among various rs-fMRI parameters, both the amplitude of low-frequency fluctuations (ALFF) and the fractional ALFF (fALFF) of the BOLD signal measure the magnitude of the regional activity amplitude and reflect the intensity of spontaneous neural activity^6^. In addition, fALFF was originally regarded as less sensitive to physiological noise^7^. Regional homogeneity (ReHo) measures Kendall’s coefficient concordance in neighboring voxels to reflect the coherence of the BOLD signal amplitude between a single voxel and its nearest neighbors^8^. Given the computational basis of this parameter, it has been suggested as a measure of localized connectivity^9^, providing information about local alterations in brain function. Functional connectivity strength (FCS), also known as degree centrality, takes a given region’s relationship with the entire functional connectome into account. Examinations of FCS have focused on the identification of “functional hubs” in whole-brain networks^10,11^. Unlike the functional connectivity approach, all of these parameters do not require a priori seed selection; therefore, they have the potential to evaluate abnormalities of certain brain regions at the whole-brain level.

Previous studies have successfully revealed altered ALFF^12–14^, fALFF^15–17^, ReHo^18,19^, and FCS^20,21^ maps in various cerebral regions, including traditional CSTC circuits and newly found regions, such as the parietal, occipital, and temporal lobes and the cerebellum. These altered spontaneous neuronal activity and OCD-related brain network hubs improve our understanding of the pathophysiologic characteristics of OCD by revealing intrinsically abnormal function within and beyond CSTC circuits. However, previous studies mainly focused on localizing alterations based on group**-**level differences using univariate analysis and ignored information contained in spatial distribution patterns. Thus, whether group differences can be useful for informing the diagnosis of individual OCD patients remains unclear.

Recently, multivariate pattern analysis based on a machine learning algorithm has been introduced for neuroimaging analysis. It is a promising analytical technique allowing the classification of individual observations into distinct groups and is sensitive to spatially distributed information. This approach has been used to identify neural imaging biomarkers of psychiatric disorders based on both structural and functional images^22,23^. In particular, rs-fMRI has been applied to classify autism^24^, depression^25^, and schizophrenia^26^ with moderate accuracy using the support vector machine (SVM), a multivariate pattern analysis-based classifier. However, in regard to OCD, although accurate classification has been achieved based on structural^27^, diffusion^28^, and task-based functional MRI images^29^, few studies have explored the potential diagnostic value of rs-fMRI data to date, with one study reporting 73% classification accuracy by a whole-brain functional connectivity network^30^. However, it is difficult to draw a conclusion about the classification value of certain abnormal regions from such a study.

In the current study, we employed a multiparameter classification approach to distinguish drug-naive patients with OCD from healthy control subjects (HCS) at the individual level based on intrinsic neural activities reflected by ALFF, fALFF, ReHo, and FCS. Our aims were, first, to investigate which rs-fMRI parameter achieves the best discrimination between OCD and HCS and, second, to examine whether there is overlap between multivariate pattern analysis and univariate analysis.

## Materials and methods

### Subjects

This retrospective study was approved by the Ethics Committee of the West China Hospital, Sichuan University, and written informed consent was obtained from each participant. A total of 54 OCD patients and 54 sex- and age-matched HCS participated in this study (Table 1). All participants were right-handed and native Chinese speakers. OCD patients were recruited from the Mental Health Center, West China Hospital, Sichuan University, and diagnoses were confirmed using the Structured Clinical Interview for DSM-IV Axis I disorders (SCID) by two experienced psychiatrists. Exclusion criteria included (1) participants younger than 18 years or older than 60 years; (2) psychiatric comorbidity assessed using the SCID; (3) any history of major physical illness, cardiovascular disease or psychiatric or neurological disorder; (4) substance abuse or dependence; and (5) pregnancy. The Yale-Brown Obsessive-Compulsive Scale (Y-BOCS) was used to rate the severity of OCD symptoms, and the 14-item Hamilton Anxiety Scale (HAMA) and 17-item Hamilton Depression Scale (HAMD) were used to rate anxiety and depressive symptoms, respectively.

**Table 1:** Demographic and clinical characteristics of the participants

Variable	OCD (*n* = 54)		HCS (*n* = 54)		*p*-value
	Mean	SD	Mean	SD	
Gender (male/female)	34/20	-	34/20	-	1.00
Age (years)	30.41	8.07	28.39	11.22	0.29
Course of illness (years)	8.15	5.69	-	-	
Y-BOCS			-	-	
Obsession score	10.67	3.60	-	-	
Compulsive score	10.06	4.44	-	-	
Total score	20.72	5.30	-	-	
HAMD 17	8.19	5.87	-	-	
HAMA 14	9.24	5.15	-	-	

*OCD* obsessive-compulsive disorder, *HCS* healthy control subjects, *SD* standard deviation, *Y-BOCS* Yale-Brown Obsessive-Compulsive Scale, *HAMD* Hamilton Depression Rating Scale, *HAMA* Hamilton Anxiety Rating Scale

These patients had not received any prior psychiatric medications for various reasons, mainly because of (1) a lack of understanding or recognition of the severity of mental illness, (2) poor socioeconomic conditions that limited travel and funds for medical care in rural areas, and (3) a lack of medical care close to the time of illness onset due to the limited popularity of family physicians in China. As a result of these factors, each patient had been sheltered in the home without medical care through the course of the illness.

HCS were recruited from the local area using poster advertisements and were screened using the SCID (nonpatient edition) by the same psychiatrists to confirm the current absence of psychiatric and neurological illness, as well as the absence of a history of psychiatric illness among first-degree relatives.

### Image acquisition

The MRI examinations were performed via a 3-Tesla GE MRI system with an 8-channel phase-array head coil. Foam pads were used to reduce head motion and scanner noise. Prior to the scan, the subjects were instructed to keep their eyes closed, remain relaxed but not fall asleep, and move as little as possible during scanning. The images were obtained via a gradient-echo echo-planar imaging sequence with the following parameters: time repetition = 2000 ms, time echo = 30 ms, flip angle = 90°, slice thickness = 5 mm with no slice gap, field of view = 240 × 240 mm^2^, 30 axial slices, and 200 volumes in each run.

### Image preprocessing

The rs-fMRI data were processed using the Data Processing Assistant for Resting-State fMRI (DPARSF, http://www.restfmri.net, version 2.1), implemented within the MATLAB toolbox. We discarded the first ten time-points to ensure signal stabilization. Slice timing and head motion correction were conducted. We used the motion correction strategy suggested by Yan et al.^31^: (1) regression of realigned data on 6 head motion parameters, 6 head motion parameters one time point before, and the 12 corresponding squared items (Friston 24-parameter model)^32^ and (2) identification of “bad” time-points using a threshold of framewise displacement > 0.2 mm, as well as one back and two forward neighbors, as reported by Power et al.^33^, followed by modeling each “bad” time point as a separate regressor in the regression models^34,35^. All subjects were under the threshold of framewise displacement = 0.2 mm. The subsequent analysis was performed within good data. Next, the images were normalized to the standard Montreal Neurological Institute template and spatially resampled to a voxel size of 3 × 3 × 3 mm^3^. Subsequently, the linear trend of the fMRI data was removed, and bandpass filtering (0.01–0.08 Hz) was conducted to decrease the impact of high-frequency physiological noise and very low-frequency drift. Six motion parameters and the signals from the cerebrospinal fluid and white matter were used as nuisance covariates to reduce the effects of head motion and nonneuronal BOLD fluctuations. The Resting-State fMRI Data Analysis Toolkit (REST) (http://www.restfmri.net/forum, version 1.8) was then used for computation of ALFF, fALFF, ReHo, and FCS. Details about the calculation of the four rs-fMRI parameters are presented below.

### ALFF and fALFF calculation

The ALFF images were computed by extracting power spectra via a Fast Fourier Transform and computing the sum of amplitudes in the low-frequency bands (0.01–0.08 Hz). The ALFF measure at each voxel represents the averaged square root of the power in the above frequency windows normalized by the mean within-brain ALFF value for that subject. For fALFF, the measure was scaled by total power across all available frequencies^36^. Finally, both ALFF and fALFF images were smoothed by an 8-mm full-width half maximum (FWHM) Gaussian kernel.

### ReHo calculation

Individual ReHo maps were generated by calculating the Kendall coefficient of concordance (KCC) of the time series of a given voxel with those of its neighbors (26 voxels) in a voxelwise manner^8^. Afterwards, a whole-brain mask was adopted to remove the nonbrain tissues. For standardization purposes, the individual ReHo maps were divided by their own global mean KCC within the whole-brain mask. Then, spatial smoothing was performed on the standardized individual ReHo maps with a Gaussian kernel of 8-mm FWHM.

### FCS calculation

We first computed Pearson’s correlations between the time series of all pairs of voxels, constructing a whole-brain connectivity matrix for each participant. A prior gray matter map (threshold of 0.2) in SPM8 was employed. To improve normality, we then transformed individual correlation matrices to a *z*-score matrix using a Fisher r-to-z transformation. For a given voxel, FCS was computed as the sum of the connections (*z*-values) between a given voxel and all other voxels. Considering the ambiguous interpretation of negative correlations with removal of the global signal, we conservatively restricted our analysis to positive correlations above a threshold of *r* = 0.2. Such a threshold was chosen to eliminate voxels with weak correlations attributable to signal noise^37,38^. The FCS maps were further smoothed with an 8-mm Gaussian kernel and normalized to standard *z*-scores.

### Univariate analysis of group comparisons

To detect group differences in demographic variables between patients with OCD and HC, two-sample *t*-tests and chi-square analyses were performed using SPSS software (IBM SPSS Statistics for Windows, version 19.0). We used a univariate approach to investigate differences in ALFF, fALFF, ReHo, and FCS between OCD patients and HCS in SPM8 software (http://www.fil.ion.ucl.ac.uk/spm/software/spm8/). Statistical inferences were made at *p* < 0.05 (corrected for multiple comparisons using the false discovery rate at the voxel level).

### Multivariate pattern analysis

We used the SVM, as implemented in the PROBID software package (http://www.brainmap.co.uk/probid.htm, version 1.04), to investigate the diagnostic potential of four maps. The PROBID software allows for a linear kernel matrix (which measures the similarity between all pairs of brain images) to be precomputed and supplied to the classifier. This approach affords a substantial increase in computational efficiency and permits whole-brain classification without requiring explicit dimensionality reduction. Individual brain scans were treated as points located in a high-dimensional space defined by the rs-fMRI maps in the preprocessed images. In this high-dimensional space, a linear decision boundary was defined by a “hyperplane” that separated the individual brain scans according to a class label (in this case, OCD vs. HCS).

SVM classification aims to classify data points by maximizing the margin between classes in a high-dimensional space. This classification process consists of two steps, training and testing. First, an SVM algorithm is trained on a well-characterized sample to establish the hyperplane in high-dimensional space that best distinguishes the different categories (i.e., OCD vs. HCS). Second, once the optimal hyperplane is developed from the training data, it is applied to a new “testing” dataset to establish its generalizability. Feature selection was performed based on the training dataset. Four types of features, ALFF, fALFF, ReHo, and FCS, were used in the present study. The machine learning algorithm finds the discriminating regions using whole-brain information without prior selection of regions.

A “leave-one-out” cross validation was used, which involved excluding a single subject from each group and training the classifier using the remaining subjects. The subject pair excluded was then used to test the ability of the classifier to reliably distinguish between categories (i.e., OCD vs. HCS). This procedure was repeated for each subject pair to assess the overall accuracy of the SVM^39,40^. The statistical significance of the overall classification accuracy was determined by permutation testing^39^, a nonparametric test that involves repeating the classification procedure 1000 times with a different random permutation of the training group labels and counting the number of permutations achieving higher sensitivity and specificity than the true labels.

To enable the visualization of the discriminating pattern for each measurement, we colored all voxels that had values >30% of the maximum value of the discrimination map. This arbitrary threshold predominantly eliminates noise components, thus enabling better visualization of the most discriminating regions^41,42^. Classification performance was assessed by computing the accuracy, sensitivity, specificity, and receiver operating characteristic (ROC) curve, from which the area under the ROC curve (AUC) was calculated.

### Correlation with symptom severity

Relationships with symptom severity were examined by extracting ALFF, fALFF, ReHo, and FCS values from regions showing group differences and correlating these values with Y-BOCS scores, HAMA scores, and HAMD scores, with age, gender and onset time as covariates in the OCD group.

## Results

### Demographic and clinical characteristics

Age and gender were not significantly different between the OCD and HCS groups (*p* > 0.05). For the 54 OCD patients, the total Y-BOCS was 20.72 ± 5.30, corresponding to moderate and severe OCD symptoms, with obsessive and compulsive subscale scores of 10.67 ± 3.60 and 10.06 ± 4.44, respectively. The estimated duration of OCD symptoms was 8.15 ± 5.69 years. The HAMA score was 9.24 ± 5.15, generally accepted as normal, and the HAMD score was 8.19 ± 5.87, indicating mild depression. See Table 1 for details.

### Group comparison by voxelwise univariate analysis

**Fig. 1:**
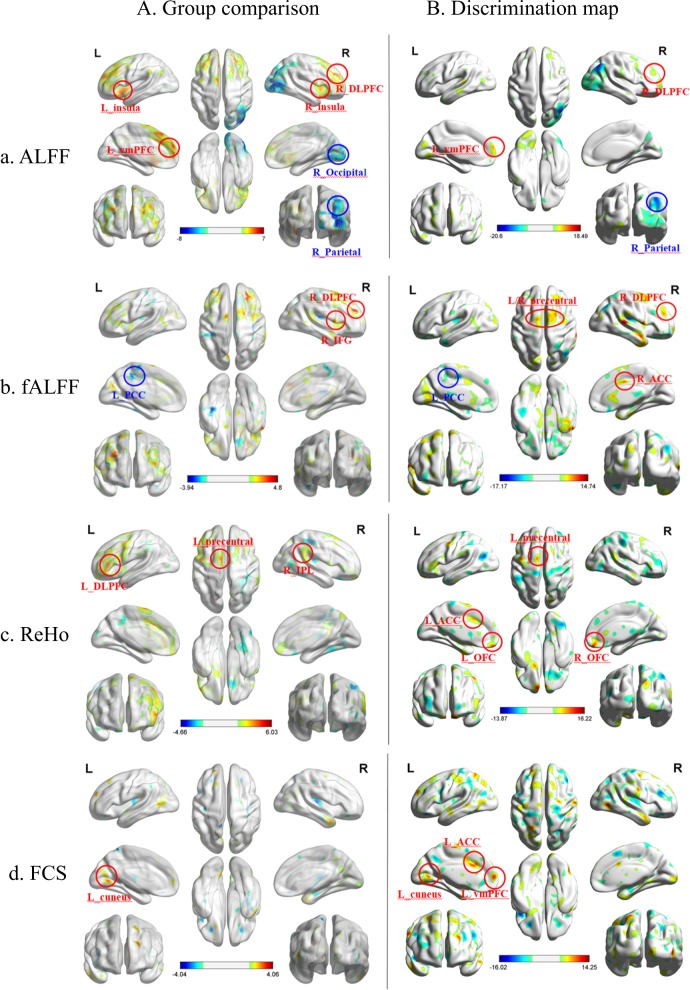
Significant regions in group comparison and discrimination map. **a** ALFF, **b** fALFF, **c** ReHo, and **d** FCS. In group comparison panel, warm colors indicate regions showing higher values in the OCD group than in HCS and cool colors indicate regions showing lower values. The color bar indicates the *T*-score. Threshold: *p* < 0.05, false discovery rate-corrected at cluster level (**a**), *p* < 0.005, uncorrected at pixel level (**b**, **c**, **d**). In discrimination map panel, warm colors indicate higher values for the parameter in OCD group than in HCS, whereas cool colors indicate higher values for the parameter in HCS than in patients with OCD. The color bar indicates the weighted vector value determined from SVM. OCD obsessive-compulsive disorder, HCS healthy control subjects, SVM support vector machine, ALFF amplitude of low-frequency fluctuations, fALFF fractional amplitude of low-frequency fluctuations, ReHo regional homogeneity, FCS functional connectivity strength

Compared with the HCS, patients with OCD had significantly increased ALFF values in the left ventral medial prefrontal cortex (vmPFC), right dorsal lateral prefrontal cortex and bilateral insula; conversely, OCD patients showed lower ALFF values in the right inferior parietal lobe (IPL), occipital lobe at a threshold of *p* < 0.05 (false discovery rate-corrected at cluster level). Although significant differences in fALFF, ReHo, or FCS values were not observed, uncorrected results are provided (*p* < 0.005, uncorrected at pixel level) (Fig. 1A).

### Classifier performance

**Fig. 2:**
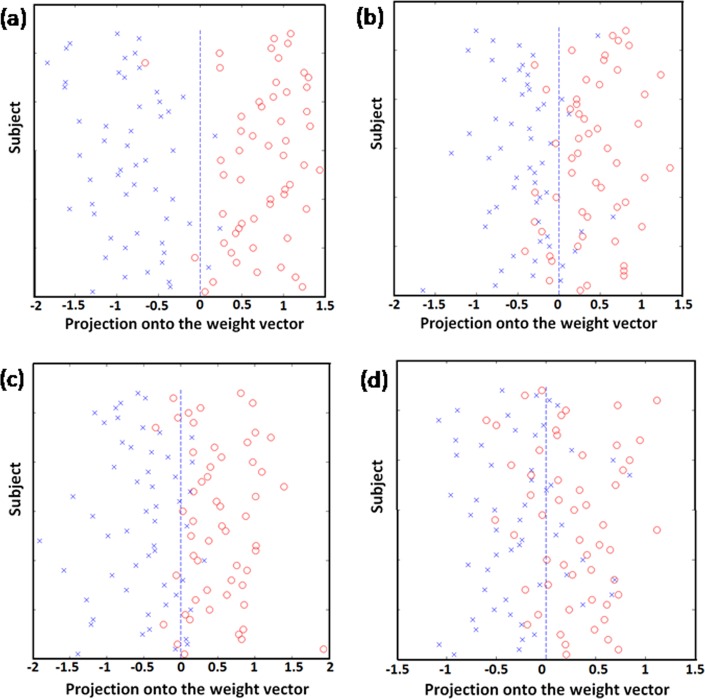
Classification plots for the SVM classifier utilizing ALFF, fALFF, ReHo, and FCS. **a** ALFF; **b** fALFF; **c** ReHo; **d** FCS (“open circle“ represents patients with OCD, and “ letter × “ represents HCS). SVM support vector machine, ALFF amplitude of low-frequency fluctuations, fALFF fractional amplitude of low-frequency fluctuations, ReHo regional homogeneity, FCS functional connectivity strength, OCD obsessive-compulsive disorder, HCS healthy control subjects

**Fig. 3:**
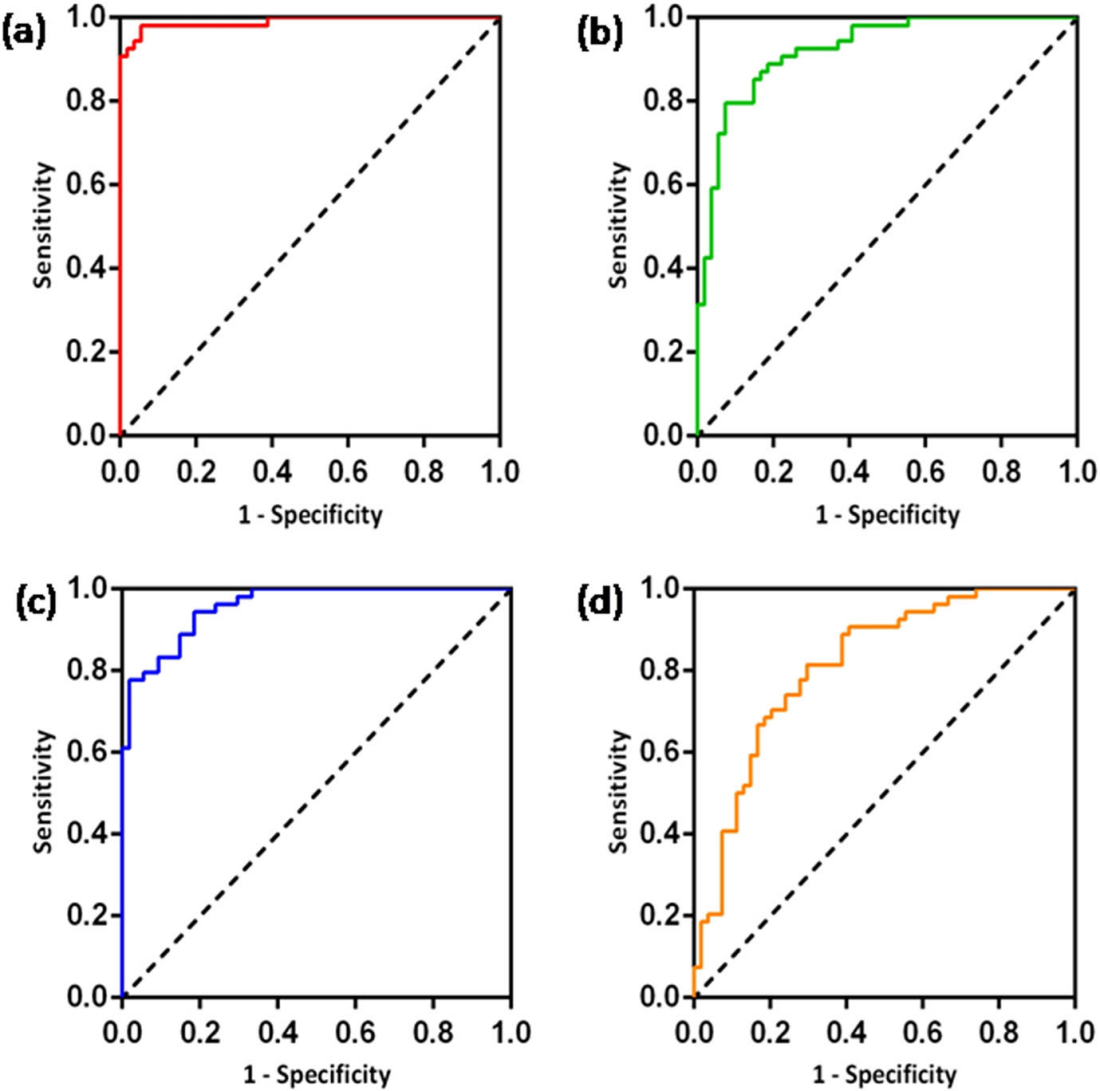
ROC curves assessing SVM performance using ALFF, fALFF, ReHo, and FCS. **a** ALFF; **b** fALFF; **c** ReHo; **d** FCS. ROC receiver operating characteristic, SVM support vector machine, ALFF amplitude of low-frequency fluctuations, fALFF fractional amplitude of low-frequency fluctuations, ReHo regional homogeneity, FCS functional connectivity strength

Figure 2 shows the results of the SVM classification between 54 OCD patients and 54 HCS based on ALFF, fALFF, ReHo, and FCS maps. The best discrimination was obtained when ALFF measures were used for accuracy, sensitivity, and specificity, with values as high as 95.37%, 96.30%, and 94.44% (*p* < 0.001), respectively, followed by the classification based on ReHo (accuracy 86.11%, sensitivity 88.89%, and specificity 83.33%, *p* < 0.001). For the fALFF measures, the accuracy was 82.41%, the sensitivity was 79.63%, and the specificity was 85.19% (*p* < 0.001). For the FCS measures, the accuracy was 74.07%, the sensitivity was 74.07%, and the specificity was 74.07% (*p* < 0.001). The ROC curves demonstrated good performance, with AUC values ranging from 0.81 to 0.99 (*p* < 0.001) (Fig. 3). An overview of the accuracy and AUC values is presented in Table 2.

**Table 2:** Accuracy and AUC values for the four parameters

Parameter	ALFF	fALFF	ReHo	FCS
Accuracy (%)	95.37	82.41	86.11	74.07
Sensitivity (%)	96.30	79.63	88.89	74.07
Specificity (%)	94.44	85.19	85.33	74.07
AUC	0.99	0.92	0.96	0.81

*ALFF* amplitude of low-frequency fluctuations, *fALFF* fractional amplitude of low-frequency fluctuations, *ReHo* regional homogeneity, *FCS* functional connectivity strength, *AUC* area under the ROC curve

*p* < 0.001

### Discrimination map of OCD abnormalities

The spatial maps of the brain regions that strongly contributed to the discrimination between patients with OCD and controls are shown in Fig. 1B, and the detailed location and information appear in Supplementary Table S1–S4. The spatial distribution of the weighted vectors can be thought of as a spatial representation of the decision boundary and thus represent a map of the most discriminating regions.

The regions that contributed to the identification of patients with OCD (OCD > HCS) in the ALFF discrimination map included the left vmPFC, right dorsal lateral prefrontal cortex and bilateral insula. In contrast, regions that contributed to the identification of controls (HCS > OCD) were mainly located in the right IPL and occipital lobe. The discriminative pattern for OCD in the fALFF map was composed of the right superior frontal lobe, bilateral precentral gyrus, right superior temporal gyrus, anterior cingulate cortex (ACC), and left cuneus and left lingual gyrus, whereas regions that contributed to the identification of HCS were mainly located in the right IPL. The discriminative pattern for OCD in the ReHo map primarily consisted of the bilateral orbitofrontal cortex (OFC), left ACC, left putamen, and left precentral gyrus. The discriminative pattern for OCD in the FCS map mainly contained the bilateral superior frontal lobe, left vmPFC, left ACC, left superior parietal, bilateral lingual gyrus, and right putamen.

### Relationships with symptom severity

No significant association was observed between the values of rs-fMRI parameters and symptom scores after corrections.

## Discussion

To our knowledge, this is the first study to investigate the potential diagnostic value of different resting-state fMRI features in adult drug-naive OCD patients. The regional neural activity across the whole-brain reflected by ALFF, fALFF, ReHo, and FCS can distinguish patients with OCD from HCS. Excellent performance was achieved when ALFF maps were employed, good performance was achieved by using ReHo maps, weaker performance was achieved by using fALFF maps, and fair performance was achieved by using FCS maps. Remarkably, the discrimination pattern of ALFF partially overlapped with the group differences. In addition, regions that contributed to the identification of patients with OCD were not only limited to the CSTC circuits, such as the vmPFC and putamen, but also involved additional brain systems, including the precentral gyrus and occipital lobe. These patterns provide preliminary support for the use of the four rs-fMRI parameters, especially ALFF, as promising classification markers for drug-naive patients with OCD.

Classification accuracy varied among different resting-state functional parameters. The four indices of spontaneous brain activity revealed increased and decreased weighted vector values in drug-naive adults with OCD compared to those of HCS, but these patterns cannot be explained as neuronal activity increases or decreases in one group relative to the other. We emphasize that due to the multivariate character of SVM, each region in the discrimination maps should be interpreted in the context of the entire discriminating pattern and should not be considered in isolation. In multivariate methods, an individual region may display high discriminative power for two possible reasons: (i) the presence of a large group difference in that region; or (ii) the region is highly intercorrelated with other regions of the network. Thus, discrimination maps should be interpreted as spatially distributed patterns rather than as individual regions^43^.

Of the four measurements, ALFF showed the greatest diagnostic accuracy for discriminating patients with OCD from HCS. It has been shown that ALFF directly correlates with the intensity of spontaneous neural activity in the resting state and is related to the rate of regional glucose metabolism^44^. Nugent et al. suggested that impaired glutamate cycling is widespread throughout the cortex, particularly implicating neuronal dysfunction. This effect could make ALFF more sensitive to detecting dysfunctional neural activity than the other three parameters. In addition, we found that regions showing significant group differences had partially overlapping discrimination patterns. The strong group differences for ALFF may underlie the excellent classification achieved in the current study. Therefore, the SVM enabled the identification of brain regions that corroborate the existing differences in ALFF between patients with OCD and HCS, providing support for ALFF as a promising classification marker for OCD.

Initially, scholars believed that fALFF selectively suppressed artifacts from nonspecific brain areas while enhancing signals from cortical regions associated with brain activity, making use of the distinct characteristics of signals in the frequency domain and would therefore significantly improve the sensitivity and specificity in detecting regional spontaneous brain activity compared with ALFF^7^. However, in the present study, a very different profile of spatially distributed patterns was observed in the fALFF map, with generally lower classification accuracies than those of ALFF. Therefore, we supposed that in the fALFF approach, power spectrum fractionalizing resulted in suppressed power in the low-frequency range in regions such as the cisterns, ventricles and sagittal sinus, as well as altered spectral distribution. Thus, fALFF was incapable of detecting subtle information for optimal differentiation.

ReHo showed good classification performance as well, particularly in the bilateral OFC, left ACC, precentral gyrus and putamen, although the comparison between the two groups was not significant. Since the discrimination is based on the whole-brain pattern by taking into account correlations among the regions, rather than evaluating individual regions, we suppose that the whole-brain spatial pattern of ReHo differs between OCD and HCS. While the different resting-state fMRI approaches mentioned above are promising for measuring intrinsic spontaneous brain activity, we used graph-based voxelwise FCS to reveal the value of hub-related abnormalities in discrimination OCD. In general, regions with higher FCS values usually suggest a central role in the functional integrity of the whole-brain networks^21^. The automated classification of patients with OCD versus HC using FCS did not show high accuracy. The reason for this may lie in the fact that the range of FCS values was highly overlapping for the two groups. Therefore, both weak group differences and poor classification were achieved.

The discrimination regions identified in our study are within and beyond CSTC circuits. Previous neuroimaging studies have revealed the critical role the ACC/vmPFC, a region crucially involved in detecting the presence of cognitive conflicts, error monitoring and detection^2^, in OCD. A recent meta-analysis study^4^ has shown that the gray matter volume was reduced in ACC/vmPFC in patients with OCD. In the task state, several studies^45,46^ reported that increased ACC activation in patients with OCD in relation to error processing correlated with disease severity. In the resting state, Tian et al.^20^ and Hou et al.^21^ reported increased functional connectivity strength in the ACC in OCD patients compared with HCS. The hyperactivity in the ACC may reflect dysfunction of the action monitoring system and result in abnormal symptoms of OCD, such as feelings of being erroneous, a constant need for correction and incomplete performance. OCD patients also exhibited greater activity in the vmPFC due to a failure to deactivate this DMN region^47^, perhaps reflecting an inability of patients to disengage from automatic evaluative processes when errors occur. In addition, meta-analytical^4^ findings have also reported that structurally and functionally overlapping regions in the putamen show increases in both gray matter volume and activity in patients with OCD. Furthermore, Beucke et al.^48^ found greater local connectivity in the OFC and the putamen, and this connectivity was positively correlated with OCD symptom severity. Consistent with these findings, the ACC/vmPFC and putamen displayed a high degree of discriminative ability between OCD patients and healthy controls in the present study, providing further support for dysfunction in CSTC pathways in patients with OCD.

In addition, there is some evidence suggesting that individuals with OCD show activation in regions within the sensorimotor network, including the precentral gyrus and supplementary area, in inhibitory control processes^49,50^, which partially explains the nature of inhibitory control deficits in OCD. Furthermore, some results demonstrated that unmedicated OCD patients have impaired sensory-motor integration and sensory gating, as measured by prepulse inhibition and transcranial magnetic stimulation, which indicates that the abnormality of the sensorimotor system might impair the ability of OCD patients to suppress internally triggered intrusive and repetitive movements and thoughts^51,52^. Therefore, the notable contribution of these regions to accurate discrimination in the present study provides further support for their involvement in OCD.

Our finding that the occipital lobe contributed to the discrimination of OCD patients from HCS is consistent with other evidence implicating the occipital region in OCD. For instance, OCD patients with poor insight had increased ALFF in the right middle occipital gyrus^53^. A previous study^54^ reported that OCD patients showed hypoactivation of the superior and inferior occipital cortex during a target detection task following negative internally focused attention states, pointing to an OCD-related impairment in the visual processing of external stimuli when patients have experienced a period of negative internal focus.

It is noteworthy that there are some limitations of the present study. First, although the findings were encouraging, the sample size was relatively small, and the generalizability of the results was, therefore, unclear. Larger sample sizes are needed to confirm our findings. Second, we separated only patients with OCD from healthy subjects, leaving an unresolved issue of whether the use of the SVM to rs-fMRI would also successfully discriminate between subtypes of OCD. Future studies may be dedicated to the differential diagnosis of patients with OCD across subtypes or to differentiating between patients with OCD and other psychiatric conditions with potentially overlapping symptoms. Third, we need to be cautious when explaining the results of ALFF, as it contains not only low-frequency neuronal fluctuations but also low-frequency physiological fluctuations, such as breathing pattern changes. Given the low sampling rate used in this study (TR = 2 s), we cannot fully exclude the confounding effect of cardiac pulsation and respiratory effects.

In conclusion, our results provide preliminary support for the hypothesis that multiple rs-fMRI features can be utilized for the diagnostic classification of drug-naive patients with OCD, with the ALFF providing the greatest accuracy. Furthermore, our findings emphasize the role of regions within and outside CSTC circuits in the pathophysiology of OCD. Therefore, this study demonstrates that the application of supervised machine learning methods, such as SVM, to neuroimaging data could potentially be used for reliable OCD classification, and also adds the development of psychoradiology^55^ that applies imaging to psychiatry and psychology.

## Supplementary information

Supporting Information for Investigating the predictive value of different resting-state functional MRI parameters in obsessive-compulsive disorder

Certificate of Nature Research Editing
