# Supplementary material for: Investigating the predictive value of different resting-state functional MRI parameters in obsessive-compulsive disorder
Source: Transl Psychiatry. 2019 Jan 17;9:17. doi: 10.1038/s41398-018-0362-9 (PMC6336781; doi:10.1038/s41398-018-0362-9)
Supplement: Supplementary file 1 — Supporting Information for Investigating the predictive value of different resting-state functional MRI parameters in obsessive-compulsive disorder [file 41398_2018_362_MOESM1_ESM.doc]

*Supporting Information for Investigating the predictive value of different resting-state functional MRI parameters in obsessive-compulsive disorder*

**Table S1 Regions displaying high discrimination weights between patients with OCD and controls for ALFF measures**

| Brain region | MNI coordinate | | | wi |
| --- | --- | --- | --- | --- |
|  | x | y | z |  |
| **OCD>HC** | | | | |
| Frontal | | | | |
| R DLPFC | 3 | 57 | -8 | 11.85 |
| R superior frontal | 29 | 53 | 36 | 14.73 |
| L vmPFC | -37 | 53 | 22 | 14.16 |
| Occipital | | | | |
| L lingual | -37 | -65 | -17 | 12.86 |
| L cuneus | -2 | -79 | 20 | 9.82 |
| Temporal | | | | |
| L superior temporal | -46 | 19 | -17 | 9.97 |
| L middle temporal | -46 | -74 | 20 | 10.69 |
| Subcortical |  |  |  |  |
| L insular | -50 | 23 | 1 | 7.34 |
| R insular | 53 | 20 | 1 | 13.00 |
| **OCD<HC** | | | | |
| Parietal | | | | |
| R inferior parietal | 44 | -65 | 48 | -19.47 |
| R angular | 49 | -65 | 36 | -14.65 |
| Occipital | | | | |
| R middle occipital | 46 | -77 | -8 | -12.07 |
| R superior occipital | 32 | -88 | 20 | -8.37 |
| R lingual | 17 | -94 | -8 | -8.85 |
| R cuneus | 13 | -100 | 2 | -12.55 |
| R fusiform | 38 | -81 | 17 | -17.70 |

Table S2 Regions displaying high discrimination weights between patients with OCD and controls for fALFF measures

| Brain region | MNI coordinate | | | wi |
| --- | --- | --- | --- | --- |
|  | x | y | z |  |
| **OCD>HC** | | | | |
| Frontal | | | | |
| L precentral | -60 | -24 | 23 | 10.79 |
| R precentral | 59 | 4 | 8 | 12.08 |
| R superior frontal | 25 | 42 | 22 | 11.76 |
| R middle frontal | 43 | 1 | 55 | 9.11 |
| Occipital | | | | |
| L lingual | -37 | -83 | 23 | 9.18 |
| L cuneus | -5 | 73 | 23 | 7.81 |
| Temporal | | | | |
| R superior temporal | 41 | -10 | -34 | 9.34 |
| Subcortical | | | | |
| R dorsal anterior cingulate cortex | 7 | -2 | 40 | 10.79 |
| **OCD<HC** | | | | |
| Parietal | | | | |
| R inferior parietal | 42 | -66 | 41 | -10.5 |

Table S3 Regions displaying high discrimination weights between patients with OCD and controls for ReHo measures

| Brain region | MNI coordinate | | | wi |
| --- | --- | --- | --- | --- |
|  | x | y | z |  |
| **OCD>HC** | | | | |
| Frontal | | | | |
| L OFC | -4 | 54 | -19 | 13.65 |
| R OFC | 6 | 52 | -19 | 14.36 |
| L precentral | -8 | -3 | 67 | 10.81 |
| L inferior frontal | -48 | 24 | 5 | 8.15 |
| Parietal | | | | |
| R superior parietal | 19 | -85 | 37 | 9.57 |
| Occipital | | | | |
| R inferior occipital | 34 | -91 | -14 | 12.94 |
| Subcortical |  |  |  |  |
| L anterior cingulate | -8 | 15 | 36 | 9.21 |
| L putamen | -13 | 9 | -7 | 11.78 |
| **OCD<HC** | | | | |
| Frontal | | | | |
| R middle frontal | 48 | 47 | 10 | -8.79 |
| R precentral | 30 | -18 | 68 | -6.36 |
| Parietal | | | | |
| L postcentral | -6 | -28 | 66 | -8.13 |
| R postcentral | 51 | -23 | 51 | -9.17 |
| L inferior parietal | -48 | -36 | 49 | -8.56 |
| L precuneus | -9 | -44 | -33 | -6.09 |
| Occipital | | | | |
| L middle occipital | -12 | -97 | 13 | -6.82 |

Table S4 Regions displaying high discrimination weights between patients with OCD and controls for FCS measures

| Brain region | MNI coordinate | | | wi |
| --- | --- | --- | --- | --- |
|  | x | y | z |  |
| **OCD>HC** | | | | |
| Frontal | | | | |
| L vmPFC | -4 | 58 | 8 | 10.9 |
| L superior frontal | -20 | 53 | 29 | 10.82 |
| R superior frontal | 17 | 55 | 37 | 9.50 |
| L precentral | -54 | 1 | 6 | 8.80 |
| R precentral | 60 | -8 | 30 | 9.19 |
| Parietal | | | | |
| L superior parietal | -23 | -67 | 37 | 9.34 |
| Occipital | | | | |
| L lingual | -34 | -67 | -14 | 8.33 |
| R lingual | 27 | -75 | -16 | 7.16 |
| Temporal | | | | |
| L superior temporal | 29 | -20 | -26 | 7.47 |
| R superior temporal | 52 | 11 | -26 | 10.35 |
| Subcortical | | | | |
| L anterior cingulate cortex | -4 | 16 | 32 | 12.93 |
| R putamen | 24 | 7 | 6 | 6.79 |
| R caudate | 15 | 19 | 7 | 6.35 |
| **OCD<HC** | | | | |
| Frontal | | | | |
| R superior frontal | 45 | 36 | 37 | -11.39 |
| R middle frontal | 29 | 35 | 13 | -8.84 |
| Parietal | | | | |
| L inferior parietal | -41 | -42 | 55 | -8.64 |
| R inferior parietal | 49 | -39 | 55 | -11.46 |
| L precuneus | -5 | -49 | 43 | -9.01 |
| L postcentral | -27 | -41 | 41 | -13.64 |
| R postcentral | 26 | 19 | 67 | -9.64 |
| Temporal | | | | |
| R temporal | 55 | -36 | -24 | -7.61 |

Table S5 Accuracies for the four parameters by 2-fold and 10-fold cross validation

|  | Accuracy (%) | | Sensitivity (%) | | Specificity (%) | |
| --- | --- | --- | --- | --- | --- | --- |
|  | 2-fold CV | 10-fold CV | 2-fold CV | 10-fold CV | 2-fold CV | 10-fold CV |
| ALFF | 94.40 | **95.37** | 94.40 | **96.43** | 94.40 | **94.44** |
| fALFF | 67.60 | **82.41** | 70.40 | **83.33** | 64.80 | **81.48** |
| ReHo | 81.48 | **83.33** | 79.63 | **79.63** | 83.33 | **87.04** |
| FCS | 66.67 | **72.22** | 66.67 | **66.67** | 66.67 | **77.78** |
| p<0.001 |  |  |  |  |  |  |
